# Determinants of information security policy compliance: integrating task-technology fit and threat avoidance appraisals in the digital workplace

**DOI:** 10.3389/fpsyg.2026.1861737

**Published:** 2026-07-22

**Authors:** Mada Alassaf, Ali Alkhalifah

**Affiliations:** 1 Ministry of Education, Riyadh, Saudi Arabia; 2 Department of Information Technology, College of Computer, Qassim University, Buraidah, Saudi Arabia

**Keywords:** compliance behavior, information security policy, Information security policy compliance, task–technology fit, technology threat avoidance

## Abstract

**Introduction:**

The integrity of organizational information systems fundamentally relies on the psychological commitment of users to adhere to security protocols. Informational security policy (ISP) depends on users’ compliance to protect their organization’s information and technology assets against security threats.

**Methods:**

Drawing on the intersection of cognitive and behavioral psychology, this study examines information security policy compliance (ISPC) behavior by integrating the Task–Technology Fit (TTF) model, technology threat avoidance theory (TTAT), and the Theory of Planned Behavior (TPB). This study proposes a comprehensive research model to elucidate the cognitive factors and motivational drivers that shape an employee’s behavioral intention to comply. Utilizing a partial least squares structural equation modeling (PLS-SEM) approach, we empirically tested the model with data from 288 employees across public and private sectors in Saudi Arabia.

**Results:**

The results show that Task and technology characteristics significantly drive task-technology fit, which, along with self-efficacy, social influence, and policy effectiveness, positively predicts security compliance intentions and subsequent behavior. Conversely, compliance intention is hindered by perceived costs, while the influence of perceived security threats remains statistically insignificant. The study discusses key theoretical and applied insights, providing a roadmap for future research in the field.

## 1 Introduction

Information security breaches have become increasingly costly over time and reached a critical threshold in 2026. According to the 2026 IBM Cost of a Data Breach Report, the global average cost of a security incident has climbed to a record-breaking US$4.88 million (IBM Security, 2026). In the Middle East, while the region has seen a moderate 18% stabilization in costs due to aggressive adoption of AI-driven security automation, the average cost remains significantly high at US$7.29 million, representing the second-highest regional average in the world (IBM Security, 2026). Furthermore, the “human factor” continues to be the most pervasive vulnerability; current empirical findings indicate that approximately 68–88% of all organizational breaches involve an explicit human element, ranging from sophisticated AI-driven phishing campaigns to simple system misconfigurations (Verizon, 2026; World Economic Forum, 2026). This persistence of human-related risk underscores that modern organizational resilience depends less on hardening technical perimeters and substantially more on the psychological, volitional, and task-aligned compliance behaviors of individual enterprise users. To mitigate these systemic exposures, organizations develop comprehensive Information Security Policies (ISPs). Senior management must pay close, sustained attention to ensuring employee compliance with these policies (Moody et al., 2018).

Grounded in the peer-reviewed Information Systems (IS) literature, Information Security Policy Compliance (ISPC) is conceptualized as an employee’s deliberate socio-behavioral choice to safeguard their organization’s informational and technological assets by adhering to mandated directives (Bulgurcu et al., 2010; Ifinedo, 2014; Yazdanmehr and Wang, 2016). When employee actions are managed and guided properly, an ISP serves as an effective behavioral control; conversely, when compliance behaviors are unmonitored or structurally unsupported, the human element becomes a primary vector for organizational vulnerability (Humaidi and Balakrishnan, 2015a). Because security compliance is fundamentally an issue of human behavior, achieving consistent compliance is vital to reducing internal threats and cultivating an institutional safety culture (Hengstler et al., 2023; Hong and Furnell, 2022; Hwang and Seo, 2025). Extensive research has examined the multi-dimensional internal, socio-psychological, and external variables that motivate or deter human adherence to ISPs (Alassaf and Alkhalifah, 2021; Ali et al., 2020; Amankwa et al., 2018; Chua et al., 2018; D’Arcy and Teh, 2019; Dhillon et al., 2020; Koohang et al., 2020; Vedadi et al., 2024; Zhang and Pang, 2025).

To map these behavioral pathways, researchers have heavily deployed socio-behavioral models such as the Theory of Planned Behavior (TPB), Protection Motivation Theory (PMT), technology threat avoidance theory (TTAT), and General Deterrence Theory (GDT) (Herath and Rao, 2009; Moody et al., 2018; Sikolia et al., 2018). However, while these motivational frameworks have established a profound footprint in the ISPC literature, a critical contextual and theoretical gap persists. Prior behavioral research overwhelmingly models compliance as an abstract, independent psychological intent, treating policy adherence as a purely volitional choice. It consistently overlooks the operational reality that interacting with, executing, and adhering to security controls (e.g., managing complex passwords, classifying files, or following multi-factor authentication paths) constitutes an active workplace task that must be performed within the boundaries of the organization’s existing technology infrastructure.

Consequently, this paper addresses this gap by departing from traditional, additive socio-psychological approaches. It presents an integrated hybrid behavioral model that merges the structural logic of the Task-Technology Fit (TTF) model (Goodhue and Thompson, 1995), the cognitive appraisal logic of the technology threat avoidance theory (TTAT) (Liang and Xue, 2009), and the volitional engine of the Theory of Planned Behavior (TPB) (Ajzen, 1991). This integrated framework allows us to explain an operational phenomenon that prior, motivation-only models cannot: how the technological configuration of a workplace directly shapes, enhances, or suppresses the psychological drivers of policy compliance. For instance, we argue that structural task-system compatibility directly feeds into an employee’s cognitive appraisals—where a poor fit between work tasks and security technologies (low TTF) spikes the perceived cost of compliance and minimizes self-efficacy, ultimately degrading compliance intentions and behaviors. This positioning directly echoes recent assertions by Tejay and Winkfield (2026), who emphasize that task-oriented organizational influences and systemic work structures dictate the boundary thresholds of an employee’s behavioral compliance.

This study strives to answer the following research questions: *To what extent do cognitive threat appraisals, perceived compliance costs, and task-technology fit influence ISP compliance behaviors?* The study’s specific contributions are as follows: one of the few studies to use factors related to the technology-related behavior theories. Second, this study examines the extent to which TTF, perceived security threat, perceived cost of compliance, perceived effectiveness of ISP, self-efficacy, and social influence affect ISPC behavior. Third, this study develops and validates the TTF construct within ISPC research, with this considered to be the first such attempt in this field of research. Fourth, this study provides valuable direction for studies on ISPC, an area of study which, to date, has not been well researched in Saudi Arabia.

The remainder of this paper is organized as follows: section 2 reviews the related works, and section 3 discusses the theoretical background of ISPC and the underlying theories. Section 4 presents the research model and hypothesis development. Section 5 outlines the research methodology, section 6 explains the data analysis and results, and section 7 discusses the results. Section 8 presents the contributions, section 9 discusses the limitations and future work, and section 10 concludes the paper.

## 2 Related work

Extant behavioral research has decisively shifted focus away from purely technical perimeters toward the nuanced socio-behavioral and psychological determinants influencing policy compliance (Alassaf and Alkhalifah, 2021; Herath and Rao, 2009; Siponen et al., 2014). Within contemporary organizational psychology, information security compliance is no longer viewed as a static, reactive obligation; instead, recent literature frames steady policy adherence as a critical manifestation of organizational citizenship behavior (OCB) (Vedadi et al., 2024). This paradigm suggests that when employees internalize security as a voluntary, proactive contribution to the collective organizational safety rather than an externally imposed corporate burden, compliance behaviors become highly resilient and integrated into daily operations.

However, enforcing rigid behavioral boundaries within highly demanding digital environments frequently generates significant psychological friction. Complex or obstructive security configurations can induce severe “security stress” and “security fatigue,” driving down compliance when communication channels between management and staff are broken or unidirectional (Humaidi and Balakrishnan, 2015a; Hwang and Seo, 2025). This variance in individual psychological resilience indicates that traditional organizational deterrence mechanisms (e.g., formal sanctions or penalties) do not affect employees uniformly. Instead, the effectiveness of formal enforcement varies significantly based on an individual’s baseline compliance orientation, baseline motivations, and perceived workplace pressures (Hengstler et al., 2023; Herath and Rao, 2009; Sikolia et al., 2018).

Furthermore, an individual’s intrinsic motivation to protect enterprise data is deeply tied to the unwritten psychological contract between the employee and the organization; perceptions of a psychological contract breach heavily deplete an individual’s willingness to go through the extra effort required to maintain data safeguards (Lee et al., 2023). In modern digitized environments where threat information is highly amplified via social interactions, an employee’s protective choices are governed heavily by how privacy risks are communicated, how peer networks perceive those vulnerabilities, and how organizational structures alleviate cognitive burdens (Zhang and Pang, 2025).

## 3 Theoretical foundations

### 3.1 Task-technology fit (TTF) model

The task-technology fit (TTF) model posits that information systems utilization and performance are direct products of the structural alignment between the requirements of an individual’s work duties (Task Characteristics) and the functionalities of the systems provided (Technology Characteristics) (Goodhue and Thompson, 1995; Goodhue, 1998). Tasks represent the operational actions required to transform organizational inputs into outputs (Perrow, 2018), often characterized by non-routineness and interdependence (Goodhue and Thompson, 1995). Technology characteristics refer to system attributes such as usefulness, ease of learning, accuracy, and reliability (Goodhue and Thompson, 1995).

While the TTF framework is widely validated across diverse operational domains—including healthcare (Ali et al., 2018), higher education (Bere, 2018), finance (Tam and Oliveira, 2016), and tourism (Dedeke, 2016)—its application within behavioral information security remains scarce (Kim et al., 2020). Prior research typically combines TTF with adoption models to evaluate baseline productivity (Moreno and Cavazotte, 2015; Spies et al., 2020; Shahbaz et al., 2021).

This study departs from traditional performance-centric applications to address a critical theoretical gap: the lack of empirical dimensionality of the TTF construct within Information Security Policy Compliance (ISPC) research. This study reconceptualizes the Task as the collective compliance behaviors an employee must routinely execute (e.g., password management, data handling, incident reporting) and the Technology as the active security systems and authentication tools embedded within their digital workplace. High TTF indicates that corporate security controls align with the operational speed of work tasks, effectively mitigating systemic friction.

### 3.2 Technology threat avoidance theory (TTAT)

The technology threat avoidance theory (TTAT) explains the cognitive-calculative mechanisms driving individuals to voluntarily implement safeguards to avoid malicious information technology threats (Liang and Xue, 2009). Developed to address a lack of context-specific theorization regarding IT risks (Herath et al., 2014; Liu et al., 2020b), TTAT models a dual-appraisal cognitive process: Threat Appraisal (evaluating perceived threat susceptibility and severity) and Coping Appraisal (evaluating perceived avoidability based on the safeguard’s effectiveness, implementation costs, and individual self-efficacy) (Liang and Xue, 2009, 2010).

If an employee perceives an administrative safeguard as carrying excessive operational costs, they experience a coping failure and resort to passive, emotion-focused coping mechanisms (e.g., policy circumvention) rather than active threat avoidance (Liang and Xue, 2009). Because organizational security policies are formally designed to guide users away from malicious IT liabilities, executing an ISP constitutes an active avoidance routine (Liu et al., 2020b; Zhang and Pang, 2025). Grounded in the core components of Protection Motivation Theory (Crossler et al., 2019), TTAT provides this study with a highly rigorous framework to capture the employee’s subjective cost-benefit calculation regarding corporate security mandates.

### 3.3 Theory of planned behavior (TPB)

The theory of planned behavior (TPB) establishes that an individual’s manifest behavior is directly determined by their behavioral intention, which is predicted by three antecedents: attitude toward the behavior, subjective norms, and perceived behavioral control (Ajzen, 1991; Fishbein and Ajzen, 1975). In information security, establishing this deliberate behavioral intent is a critical prerequisite to understanding whether organizational assets are safely guarded against internal exposures (Crossler et al., 2013; Hong and Furnell, 2022; Ifinedo, 2014; Rajab and Eydgahi, 2019).

However, incorporating TPB into comprehensive multi-theory frameworks introduces severe risks of structural fragmentation and conceptual redundancy if its omnibus constructs are merely superimposed alongside domain-specific models (Siponen et al., 2014). Therefore, to ensure statistical parsimony and context specificity, this study deliberately avoids modeling the generic, abstract forms of traditional TPB variables. Instead, following established behavioral modeling paradigms (e.g., Ifinedo, 2012; Venkatesh et al., 2003), we systematically decompose and map the underlying psychological mechanics of TPB directly onto highly granular, security-specific constructs derived from the Task-Technology Fit (TTF) model and technology threat avoidance theory (TTAT). Subjective Norms are operationalized as Social Influence (Venkatesh et al., 2003) to capture peer compliance pressures and leadership mandates. Perceived Behavioral Control is unpacked into an internal capability dimension (Self-Efficacy) and an external, objective structural dimension (Task-Technology Fit). Attitude is evaluated through the instrumental cognitions of Perceived Cost (the negative evaluation of operational sacrifice) and Perceived Effectiveness (the positive evaluation of safeguard utility).

This targeted mapping captures the complete volitional engine of the TPB while anchoring it directly within the operational realities of the digital workspace, eliminating the inefficiencies of a purely additive multi-theory assembly.

## 4 Research model and hypothesis development

**FIGURE 1:**
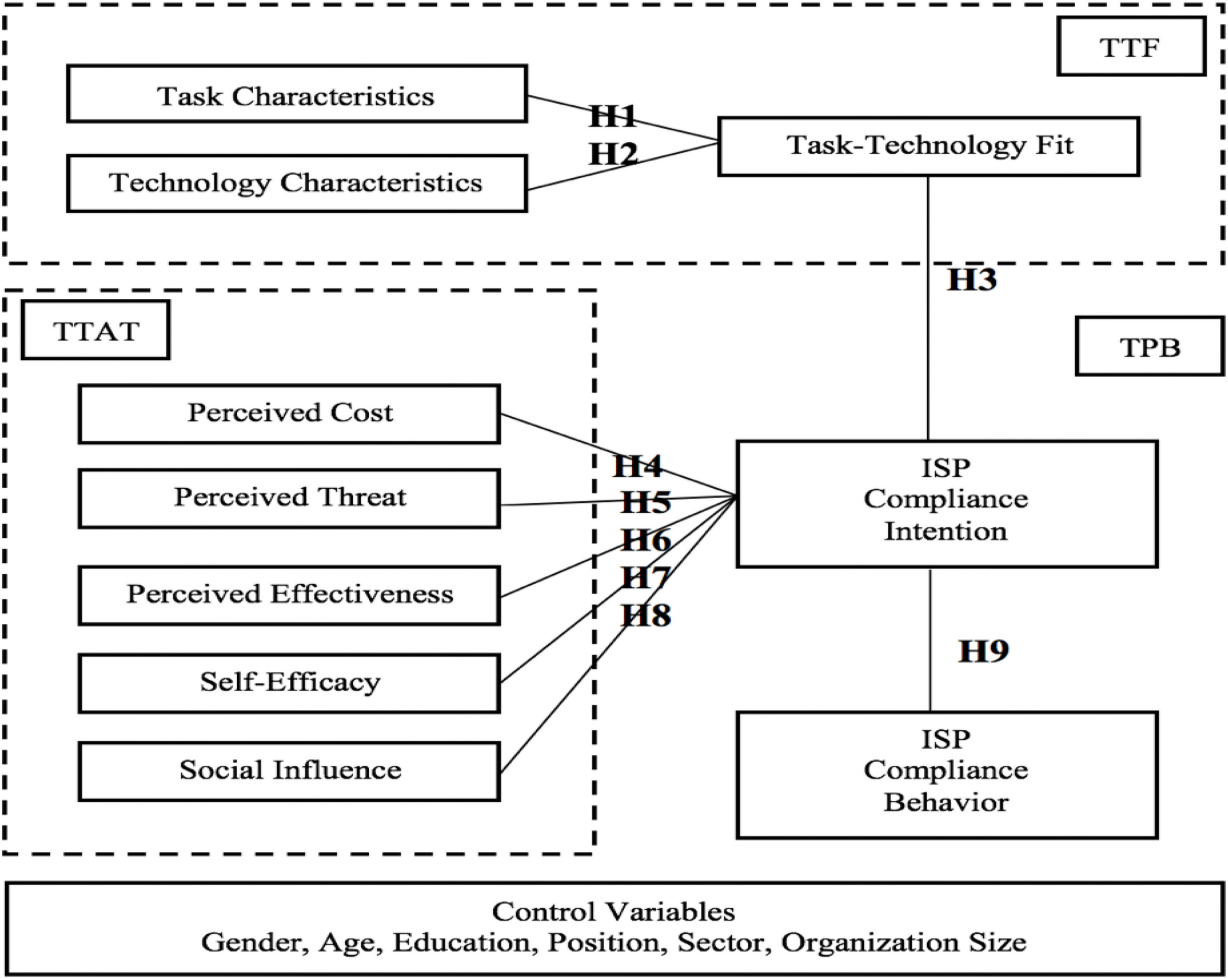
Research model.

This study assumed that task characteristics and technology characteristics influence the TTF. In addition, the TTF, perceived cost, perceived threat, perceived effectiveness, self-efficacy, and social influence were believed to affect ISPC intention based on the TTAT and the TTF model. Also, based upon the TPB, the researcher assumed that the ISPC intention affected ISPC behavior. Gender, age, education, position, sector, and organization size were used as control variables. Figure 1 shows the research model and hypotheses.

Task characteristics refer to the activities regarding the ISP in which employees engage as part of their tasks achieved using information systems (Goodhue and Thompson, 1995). The task considered in the current study is reading and understanding the ISP. The aspects of this task, that is, non-routineness and interdependence that can be achieved by information systems, are the task characteristics on which this study focuses. Tam and Oliveira (2016) argued that the individual’s intention to use information systems will be less if the task becomes more demanding, or if the technologies provide less functionality. Several studies have found a positive relationship between task characteristics and the TTF which, in turn, affects behavioral intention (Khan et al., 2018; Li et al., 2019; Lin et al., 2019; Omotayo and Haliru, 2020; Shahbaz et al., 2021). In the current context, the ISP reading and understanding characteristic significantly impacts on the TTF which, in turn, affects employees’ ISP compliance intention. Thus, the following hypothesis is proposed:

*H1*: Task characteristics have a positive influence on the task–technology fit in information security policy compliance.

Technology characteristics refer to the features of information systems used by employees in their organization that can perform specific tasks. These aspects of an information system could impact upon its utilization by employees (Tam and Oliveira, 2016). The advanced technology does not affect employees’ compliance, whereas the fit with the ISP’s demands does (Khan et al., 2018). Technology characteristics could be defined as the usefulness, ease of learning, accuracy, and reliability of the system (Omotayo and Haliru, 2020). Many studies have found a positive relationship between technology characteristics and the TTF which, in turn, affect behavioral intention (Khan et al., 2018; Lin et al., 2019; Omotayo and Haliru, 2020; Shahbaz et al., 2021). In the current context, information systems characteristics, such as availability, accessibility, and technical consulting, will assist employees with their needs for the task of reading and understanding the ISP. Thus, the second hypothesis is proposed:

*H2*: Technology characteristics have a positive influence on the task–technology fit in information security policy compliance.

The task–technology fit (TTF) determine the ability of information systems to assist employees to read and understand the ISP to support ISPC behavior. Many studies have emphasized the importance of balance between the ISP and the technologies used within the organization. O’Callaghan et al. (1992) claimed that the ISP can affect or change the technologies used inside the organization. The ISP fit should be considered when adopting or designing new technology. Choi (2009) argued that the organization with a strong ISP has achieved better incorporation of its policy-related technology. Various factors can be used to measure the level of task–technology fit (TTF), including ease of use/training, systems reliability, production timeliness, and compatibility. Li et al. (2019) found a positive relationship between TTF and behavioral intention, with a good TTF promoting behavioral intention, with the converse also being true. Therefore, the fit between ISP tasks and the information system can impact on the employee’s ISP compliance intention. Thus, the following hypothesis is proposed:

*H3*: Task–technology fit has a positive influence on information security policy compliance intention.

Perceived cost refers to the employee’s physical and cognitive efforts (time, money, energy, etc.) that are required to comply with the organization’s ISP (Liang and Xue, 2009). Past studies have considered the cost of compliance as a work impediment, with this described as a hindrance for employees’ daily functions that is caused by ISP compliance (D’Arcy and Lowry, 2019; Han et al., 2017). Employees usually analyze the cost and efforts before they decide to perform an action; therefore, these efforts could create barriers for employees, thus decreasing their behavioral motivation (Liu et al., 2020b). Some ISP requirements are time consuming and may affect employees’ productivity. For example. installing anti-virus software could require a regular update which may interrupt employees’ work or slow down information systems (Han et al., 2017). Therefore, employees could ignore some ISP procedures, if they expect that a large amount of effort will need to be executed. On the other hand, they will follow or comply with ISP procedures if only a small amount of effort is required (Kim and Han, 2019). Several studies have shown a negative association between the cost of compliance and compliance behavior (D’Arcy and Lowry, 2019; Ifinedo, 2016; Kim et al., 2014; Liu et al., 2020b; Rajab and Eydgahi, 2019; Ryutov et al., 2018). Thus, the following hypothesis is proposed:

*H4*: Perceived cost has a negative effect on information security policy compliance intention.

Perceived threat is defined as the degree to which employees perceive the security threat as a danger (Liang and Xue, 2009). Perceived threat includes perceived susceptibility and perceived severity, as explained above. Studies (Ifinedo, 2018; Liu et al., 2020a) have confirmed that employees’ high perception of information system security threat leads to their more positive attitude toward ISPC. Moreover, Koohang et al. (2021) suggested that if employees do not see that they are really confronted by security threats, they will be unlikely to adhere to the ISP’s guidelines and procedures. Yazdanmehr and Wang (2016) emphasized that employees who are less aware of security threats, and the results of their activities on themselves, others, and the organization, will not comply with their organization’s ISP easily. Past studies confirmed the positive influence of perceived threat and ISP compliance (Hina et al., 2019; Ifinedo, 2018; Liu et al., 2020a; Zhang and Pang, 2025). Therefore, the following hypothesis is formulated:

*H5*: Perceived threat has a positive effect on information security policy compliance intention.

Perceived effectiveness refers to an individual’s subjective assessment that the measures required by their organization’s ISP can lead to effective avoidance of information security threats (Liang and Xue, 2009; Liu et al., 2020b). Herath and Rao (2009) argued that if employees think about their activities and their effect on the organization’s overall information security, this will improve their security behavior. Liang and Xue (2010) believed that employees are often inclined to evaluate their organization’s information security measures, through their expected cost, their effectiveness against the security threat, and their confidence in using these measures. This concept is comparable to perceived benefits in the health beliefs model and response efficacy in the protection motivation theory, with all predicting behavior probability (Liang and Xue, 2010). Several studies have reported the positive impact of perceived effectiveness on employees’ security behavior (Ifinedo, 2012; Liang and Xue, 2010; Liu et al., 2020a; Rajab and Eydgahi, 2019). This explains the greater compliance with the ISP by employees when the ISP requirements effectively save their organization from malicious threats. Hence, the following hypothesis is proposed:

*H6*: Perceived effectiveness has a positive influence on information security policy compliance intention.

Liu et al. (2020b) described self-efficacy in the information system security context as the individual’s confidence in performing the security requirements within the ISP to safeguard the organization’s assets from different security threats. Studies have confirmed the significant relationship between self-efficacy and behavior or its intention (Huang et al., 2016). Self-efficacy has been applied in many fields, including information systems, as researchers have sought to identify the drivers of behavior (Huang et al., 2016). Humaidi and Balakrishnan (2018) suggested that increasing self-efficacy could enhance ISPC behavior, with this promoted through information security awareness programs and training. These programs can clarify the importance of information security systems and motivate individuals’ skills in the use of security measures (Humaidi and Balakrishnan, 2015b). Furthermore, Siponen et al. (2014) argued that self-efficacy regarding being able to avoid security threats considerably influences employees’ compliance intention towards policies. Many studies have highlighted the significance of self-efficacy for individuals’ security-related behavior (Huang et al., 2016; Liu et al., 2020b). In addition, past studies confirmed the positive impact of self-efficacy on ISPC (Chen et al., 2016; D’Arcy and Lowry, 2019; Humaidi and Balakrishnan, 2018; Liu et al., 2020b; Siponen et al., 2014). Therefore, the following hypothesis is proposed:

*H7*: Self-efficacy has a positive influence on information security policy compliance intention.

Social influence is defined as the degree to which employees are influenced by the perceptions of their social network members, organization, and society regarding compliance behavior (Liang and Xue, 2009). The behavior of these social groups and society could assist employees to learn and select socially desirable behaviors (Tu et al., 2015). Much of the evidence has indicated the impact of social influence on the individual’s behavior in nearly all fields, including information systems (Venkatesh and Morris, 2000). Kim et al. (2018) argued that individuals may change their behaviors and attitudes to keep up with peers and their organization, to reach their goals, to be acknowledged, or to be desirable to their social network members. Therefore, social influence makes individuals perform socially approved actions. In the current context, employees may comply with policies due to their work demands, even if they do not realize the security threats (Luarn and Huang, 2009; Hwang and Seo, 2025). Several studies have confirmed the positive effect of social influence on individuals’ behavior (Baabdullah, 2018; Tu et al., 2015). Thus, the following hypothesis is formulated:

*H8*: Social influence has a positive effect on information security policy compliance intention.

The ISP compliance intention refers to the employee’s intention to safeguard his/her organization’s information and technology assets against security threats by following their organization’s ISP (Bulgurcu et al., 2010). According to the TPB, the individual’s behavior is determined by his/her intentions to perform this behavior; therefore, when the intention to engage in such behavior is strong, the probability of performing this behavior is high (Siponen et al., 2014; Sommestad et al., 2019). This relationship has been largely proven by studies in many different fields (Ahmmadi et al., 2021; Patil et al., 2020; Si et al., 2020). Siponen et al. (2014) confirmed the need to include actual behavior in information systems research to avoid inaccurate results. In prior information systems literature, Feng et al. (2019) emphasized the significance of observing actual behavior in information systems’ security. In the current context, researches (Feng et al., 2019; Siponen et al., 2014) found a positive impact of ISP compliance intention on ISP compliance behavior. Accordingly, the current study has proposed that ISPC behavior could be widely predicted by employees’ intention to comply with their organization’s ISP. Therefore, the following hypothesis is formulated:

*H9*: Information security policy compliance intention has a positive influence on the employee’s compliance behavior with these policies.

## 5 Research methodology

To empirically validate the proposed structural model, this study deploys a cross-sectional, explanatory survey design. The subsequent sections systematically detail the quantitative sampling strategy, the target respondent distribution protocols, and the psychometric operationalization of the latent constructs utilized to evaluate the hypothesized paths.

### 5.1 Data collection and sample

This research employed a convenience sampling technique which uses non-probability sampling to collect data by selecting respondents who are convenient for researchers to access. Convenience sampling is useful as it is a speedy technique by which researchers can gather data from non-random respondents who are reachable and ready to volunteer. Convenience sampling was selected for the current study due to its flexibility in allowing the researcher to target an audience from among the appointed population, based on respondents’ accessibility and their subject matter experience (Creswell and Creswell, 2017). Moreover, this procedure was not expensive and required less of the researcher’s time.

Our target population is the employees working in either public or private organizations in Saudi Arabia. They should be dealing directly with the ISP in their organization, meaning that they were obligated to comply with this policy and the ISP should have been established previously and they should already be in the actual compliance stage. The survey was developed and distributed to respondents who met the inclusion criteria (e.g., must be employees who directly deal with the ISP in their organization). Once the final version of the survey questions was completed and approved by the Committee of Research Ethics, the researcher generated a new survey web-link from the Google form to begin the process of collecting responses.

With this online method, the link was distributed to targeted respondents through various social media platforms, such as WhatsApp work groups, as well as Twitter and Email. For this type of respondent group, the researcher contacted and achieved agreement from different employees of various departments among different government and private sector organizations to distribute the survey link to their colleagues. To encourage respondents to participate in and submit honest answers to the survey, the questionnaire was provided in both Arabic and English to avoid misunderstanding some questions. Respondents were asked to complete the questionnaires based on their experience and knowledge. At the front of the survey, the aim of the research was explained to respondents and they were informed that the data would be confidential and used only for academic purposes. After expressing their consent, the questionnaire was presented to respondents. In total, 288 respondents answered the survey questions, with the data collection process taking 1 month. The demographic profile of respondents is shown in Table 1.

**TABLE 1:** Demographic profile of respondents.

Construct	Category	Frequency	Percentage
Gender	Female	171	59.5
	Male	117	40.6
Age	Under 21 years	8	2.8
	21–30 years	132	45.8
	31–40 years	123	42.7
	41–50 years	21	7.3
	Above 50 years	4	1.4
Education	High school	11	3.8
	Diploma	32	11.1
	Bachelor’s degree	177	61.5
	Master’s degree	54	18.8
	PhD degree	13	4.5
	Other	1	0.3
Position	Senior management	21	7.3
	Middle management	47	16.3
	Specialist staff	44	15.3
	Technical	20	6.9
	Administrative	64	22.2
	Other	92	31.9
Sector	Manufacturing	13	4.5
	Telecommunications/information	21	7.3
	technology	70	24.3
	Education	24	8.3
	Health	68	23.6
	Government	30	10.4
	Finance/insurance	8	2.8
	Retail/wholesale	54	18.8
	Other		
Organization size (number of employees)	Under 10	20	6.9
	11–250	95	33.0
	251–500	43	14.9
	501–1,000	29	10.1
	1,001–5,000	57	19.8
	Above 5,000	44	15.3
		288	100

To determine the appropriate sample size, that is, the number (n) of targeted respondents, the researcher relied on the “10 times rule” (Hair et al., 2021). This rule states that the minimum sample size in the case of partial least squares-based structural equation modeling (PLS-SEM) should be equal to the larger of the following: (a) 10 times the largest number of formative indicators applied to measure a particular construct or (b) 10 times the largest number of structural relationships directed at a particular construct in the model. In the current study, the largest number of paths were directed to the ISP compliance intention. Therefore, the minimum required sample size was 50 respondents (*n* = 50). In the current study, the researcher needed to work with a larger number of respondents (*n* = 288) to collect the necessary responses for the data. Hence, this number is an acceptable effective size.

### 5.2 Construct measures

Most of the study’s scales were adopted from existing already validated scales to obtain a low measurement error (Churchill, 1979). Most of the TTAT and the TPB constructs were used in existing ISPC literature (Bulgurcu et al., 2010; Liu et al., 2020b; Siponen et al., 2014). On the other hand, items for the TTF model constructs and the social influence construct were adopted from the previous research with modifications made to ensure they were suitable for this study, with two items developed principally for this study. Therefore, they can be described as new items for analysis in this context. Specifically, items of task characteristics (TC) were adapted from Baas (2010), Goodhue and Thompson (1995), and Morgeson and Humphrey (2006); items of technology characteristics (TNC) were adapted from Goodhue (1995) and Goodhue and Thompson (1995); items of task–technology fit (TTF) were adapted from Goodhue and Thompson (1995) and Kim et al. (2020); items of perceived cost (COST), perceived threat (PT), perceived effectiveness (EFF), self-efficacy (SE), and ISP compliance behavior (CB) were adapted from Bulgurcu et al. (2010), Han et al. (2017), Liang and Xue (2010), Liu et al. (2020b), Sikolia et al. (2016), and Siponen et al. (2014); items of social influence (SI) were adapted from Venkatesh et al. (2003); and items of ISP compliance intention (CI) were adapted from Ifinedo (2012); Kim and Han (2019). All the items used responses based on a 7-point Likert scale ranging from 1 (strongly disagree) to 7 (strongly agree) (see Supplementary Appendix).

## 6 Data analysis and results

The current study chose Partial Least Squares (PLS) approach for several reasons. The PLS approach presents robustness when data are non-normally distributed. It also provides a high degree of statistical power which means that it shows the significance of relationships as they are in the population. The PLS approach is suitable for a complex model; therefore, it is preferable for the current research model which comprises nine constructs and more than 45 items (Hair et al., 2019). Moreover, the PLS approach has been widely applied in recent IS studies (Alharbi and Alkhalifah, 2025) within the ISPC context (Koohang et al., 2021; Liu et al., 2020b). We used SmartPLS 4 software (Ringle et al., 2024) to analyze the measurement and structural models.

This study utilized Partial Least Squares Structural Equation Modeling (PLS-SEM) rather than Covariance-Based SEM (CB-SEM) for three distinct reasons (Hair et al., 2019). First, this study introduces a novel structural integration by adapting and validating the multi-dimensional TTF construct within the specific behavioral domain of information security compliance. PLS-SEM is highly preferred when the primary research objective focuses on expanding or exploring new structural dimensions of established theories. Second, the structural model exhibits high complexity, incorporating 9 unique latent constructs and more than 45 distinct measurement indicators. PLS-SEM provides superior statistical power and robust estimation accuracy when handling complex path models without causing model identification constraints. Third, the goal of this research is to maximize the explained variance of the ultimate endogenous behavioral outcome (Self-Reported ISP Compliance Behavior), making the PLS causal-predictive orientation methodologically superior.

### 6.1 Measurement model

Tables 2 presents the measurement model evaluation. First, to assess the construct reliability, Hair et al. (2021) suggested 0.70 or 0.60 as minimum acceptable values for Cronbach’s alpha. However, researchers have not reached agreement on an acceptable cut-off value for Cronbach’s alpha. As stated by Taber (2018), Cronbach’s alpha values were described as satisfactory between 0.58 and 0.97, and acceptable between 0.45 and 0.98. The highest Cronbach’s alpha value of 0.936 was for scale items for “perceived effectiveness,” while the lowest value of 0.581 was for “task characteristics,” with this surpassing the satisfactory value of 0.58 as specified by Taber (2018). In psychometric analysis, Cronbach’s alpha is heavily sensitive to scale length, often underestimating internal consistency in short scales containing fewer than four indicators (Hair et al., 2021). Because our Task Characteristics scale utilizes three highly concise items designed to capture separate dimensions of work operational reality (interdependence and non-routineness), Cronbach’s alpha represents an overly conservative estimate.

**TABLE 2:** Constructs’ reliability and validity.

Construct	Items	Loading	Cronbach’s alpha	rho_A	CR	AVE
Task characteristics	TC1	0.757	0.581	0.652	0.820	0.697
	TC2	0.906				
Technology characteristics	TNC1	0.628	0.819	0.831	0.874	0.584
	TNC2	0.813				
	TNC3	0.760				
	TNC4	0.817				
	TNC5	0.787				
Task-technology fit (TTF)	TTF1	0.817	0.781	0.789	0.859	0.604
	TTF2	0.695				
	TTF3	0.793				
	TTF4	0.798				
Perceived cost	COST2	0.841	0.832	0.893	0.898	0.745
	COST3	0.918				
	COST5	0.828				
Perceived threat	PT1	0.721	0.832	0.859	0.878	0.590
	PT2	0.783				
	PT3	0.800				
	PT4	0.784				
	PT5	0.750				
Perceived effectiveness	EFF1	0.893	0.936	0.940	0.951	0.796
	EFF2	0.895				
	EFF3	0.874				
	EFF4	0.905				
	EFF5	0.893				
Self-efficacy	SE1	0.851	0.903	0.905	0.928	0.722
	SE2	0.878				
	SE3	0.877				
	SE4	0.843				
	SE5	0.797				
Social influence	SI1	0.765	0.817	0.832	0.878	0.643
	SI2	0.769				
	SI3	0.846				
	SI4	0.824				
ISP compliance intention	CI1	0.855	0.897	0.901	0.929	0.765
	CI2	0.851				
	CI3	0.913				
	CI4	0.879				
ISP compliance behavior	CB1	0.855	0.899	0.900	0.930	0.768
	CB2	0.895				
	CB3	0.878				
	CB4	0.877				

Moreover, the composite reliability (CR) values should be higher than 0.70 as suggested by Hair et al. (2021), all variables were between 0.951 and 0.820, exceeding the threshold of 0.70. Another modern indicator, namely, rho_A was also used to indicate the degree of reliability of the constructs for partial least squares analysis. A rho_A value of 0.70 or larger is preferred to demonstrate composite reliability (CR) (Dijkstra and Henseler, 2015). As presented in Table 2, the values of rho_A coefficients ranged from 0.940 for “perceived effectiveness” to 0.652 for “task characteristics.”

Second, to assess the convergent validity, the average variance extracted (AVE) value was used. An AVE value of at least 0.50 indicates sufficient convergent validity (Fornell and Larcker, 1981; Hair et al., 2021). With values ranging from 0.584 to 0.796, AVE values for all constructs were within the proposed level of being above 0.50. Moreover, the factor loading values were above 0.721, exceeding the cut-off value (over 0.7) (Hair et al., 2021). Therefore, the convergent validity is satisfied.

Third, to assess the discriminant validity, the Fornell–Larcker criterion was used. Based on the values listed in Table 3, the root value of AVE is greater than the other values; therefore, using the Fornell–Larcker criterion provides evidence of discriminant validity of each construct. Moreover, this study accessed the most recently developed measure applied to evaluate discriminant validity which is the heterotrait–monotrait (HTMT) ratio. The HTMT ratio is defined as the average of the indicators’ correlations across different constructs (heterotrait) relative to the average of the correlations of indicators within the same construct (monotrait). Henseler et al. (2015) proposed 0.90 as the acceptable threshold level of the HTMT ratio. Although “ISP compliance intention” and “technology characteristics” indicated 0.966 and 0.943, respectively, these are still considered acceptable values. Exceptionally high correlations are theoretically expected and methodologically common between conceptually adjacent, downstream variables where an intention directly dictates its corresponding behavioral endpoint (Hair et al., 2021). Henseler et al. (2015) argued that a construct’s high correlation with values close to 1 is unlikely to indicate a lack of discriminant validity, when the construct’s loading is high and the sample size is large. The results of the HTMT ratio are shown in Table 4.

**TABLE 3:** Fornell–Larcker criterion.

Construct	1	2	3	4	5	6	7	8	9	10
1. ISP compliance behavior	**0.876**	**0.875**	**0.863**	**0.892**	**0.768**	**0.850**	**0.802**	**0.835**	**0.777**	**0.764**
2. ISP compliance intention	0.872									
3. Perceived cost	-0.227	-0.232								
4. Perceived effectiveness	0.741	0.697	-0.122							
5. Perceived threat	0.415	0.436	0.212	0.561						
6. Self-efficacy	0.655	0.691	-0.154	0.714	0.476					
7. Social influence	0.670	0.676	-0.104	0.713	0.501	0.652				
8. Task characteristics	0.476	0.455	-0.048	0.493	0.412	0.453	0.414			
9. Task-technology fit (TTF)	0.484	0.504	-0.114	0.377	0.285	0.540	0.533	0.456		
10. Technology characteristics	0.375	0.382	0.006	0.288	0.237	0.407	0.445	0.460	0.756	

Diagonal elements (in bold) are the square root of the average variance extracted (AVE).

**TABLE 4:** Heterotrait–Monotrait (HTMT) ratio results.

Construct	1	2	3	4	5	6	7	8	9	10
1-ISP compliance behavior	0.966	0.258	0.131	0.587	0.514	0.744	0.594	0.645	0.943	
2-ISP compliance intention										
3-Perceived cost	0.256									
4-Perceived effectiveness	0.803	0.754								
5-Perceived threat	0.441	0.470	0.303							
6-Self-efficacy	0.719	0.759	0.168	0.769						
7-Social influence	0.765	0.774	0.158	0.804	0.577					
8-Task characteristics	0.618	0.586	0.140	0.625	0.552	0.582				
9-Task-technology fit (TTF)	0.561	0.588	0.178	0.425	0.333	0.640	0.633			
10-Technology characteristics	0.442	0.449	0.087	0.335	0.282	0.480	0.523	0.652		

### 6.2 Structural model

The evaluation metrics used to evaluate the structural model are collinearity, coefficients of determination (*R*^2^), predictive relevance (*Q*^2^), PLS Predict, and the assessment of the path coefficient. The variance inflation factor (VIF) for all constructs is considered to make sure the collinearity. The recommended value for VIF is higher than 0.20 (lower than 5) (Hair et al., 2019). All the VIF values are below 5 (CB: 2.604; CI: 2.891; COST: 1.953; EFF: 3.347; PT: 1.847; SE: 2.646; SI: 1.952; TC: 1.201; TNC: 1.724; TTF:1.572); therefore, no indication is found of collinearity issues. The *R*^2^ value presents the variance in the endogenous variable as explained by the exogenous variable(s). The *R*^2^-values of 0.75, 0.50, and 0.25 for endogenous variables, respectively, describe substantial, moderate, or weak levels of predictive accuracy (Hair et al., 2021). The model indicates 58.6% of the variance in “task–technology fit,” 62.2% of variance in “ISP compliance intention,” and 76% of variance in “ISP compliance behavior.” They can be showed as moderate and high level of predictive accuracy. The *Q*^2^ value evaluates the predictive power or predictive relevance of the endogenous constructs in the model. If Q2 values are greater than 0 (zero), this illustrates that the model has predictive relevance for endogenous constructs, while a value of 0 (zero) or less indicates a lack of predictive relevance (Hair et al., 2021). The Q2 value of “task–technology fit (TTF)” is 0.350, while for “ISP compliance intention,” it is 0.467, and for “ISP compliance behavior,” it is 0.579. All Q2 values surpassed 0 (zero), which represents a highly predictive model.

The PLS Predict examine the predictive quality of the model by comparing root mean squared error (RMSE) or mean absolute error (MAE) values of the PLS model with the values of the linear regression model (LM) (Alharbi and Alkhalifah, 2025). The distribution of error for endogenous constructs was visually interpreted as non-symmetric; therefore, the MAE was used. By comparing the MAE values of the PLS model with the MAE values of the LM, most indicators were found to score lower on the MAE in the PLS-SEM model than in the LM, as shown in Table 5. In addition, the *R*^2^ values were assessed and met Hair et al. (2021) standards. The *Q*^2^ predict values were also evaluated for the indicators and all were greater than 0 (zero). Therefore, this confirms that the structural model of this study shows medium predictive power (Hair et al., 2019).

**TABLE 5:** PLS predict results.

Item	PLS-SEM		LM	PLS-SEM MAE–LM MAE
	MAE	*Q*^2^_predict	MAE	
CB1	0.861	0.437	0.877	-0.016
CB2	0.846	0.49	0.82	0.026
CB3	0.918	0.439	0.977	-0.059
CB4	0.851	0.438	0.854	-0.003
CI1	0.829	0.488	0.814	0.015
CI4	0.872	0.433	0.9	-0.028
CI2	0.943	0.389	0.961	-0.018
CI3	0.834	0.502	0.842	-0.008
TTF1	1.03	0.311	1.012	0.018
TTF2	1.268	0.365	1.149	0.119
TTF4	1.115	0.309	1.122	-0.007
TTF5	0.941	0.398	0.936	0.005

**FIGURE 2:**
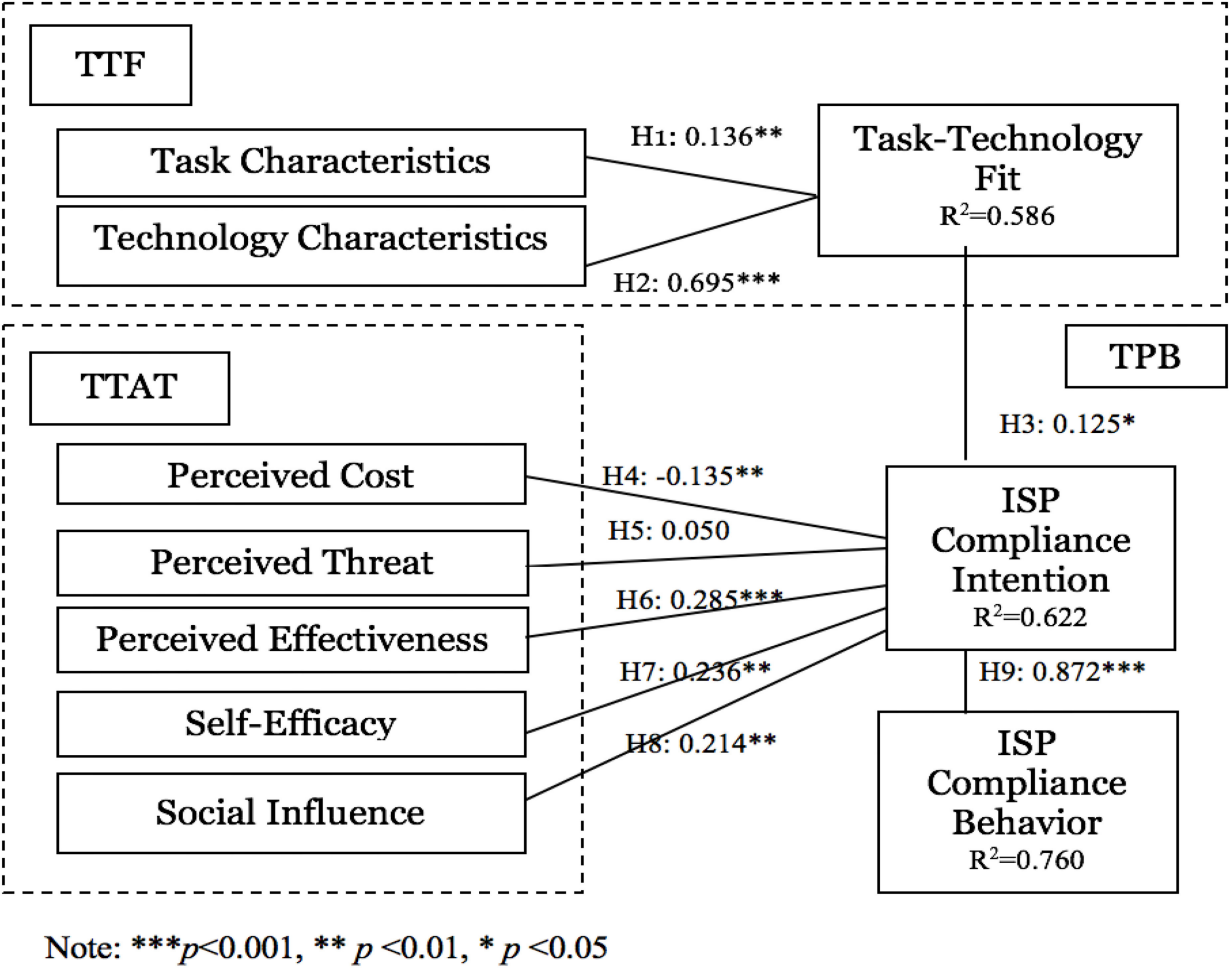
PLS test results.

The current study calculated the path coefficients that describe the hypothesized relationships connecting the constructs. This step was performed to examine the significance of the proposed hypotheses, and to analyze the relationships between the exogenous and endogenous constructs. The significance level of the path coefficient can be tested through the *p*-value and *t*-value that are estimated by employing the bootstrapping function. The analysis results are summarized in Figure 2 and Table 6.

**TABLE 6:** Path coefficients’ assessment.

H	Relationships	Original sample (O)	Sample mean (M)	Standard deviation (SD)	*t*-values	*p*-values	Decision
1	Task characteristics → task–technology fit (TTF)	0.136	0.138	0.045	2.992	0.003**	Accept
2	Technology characteristics→ task–technology fit (TTF)	0.695	0.696	0.039	17.819	0.000***	Accept
3	Task–technology fit (TTF)→ ISP compliance intention	0.125	0.123	0.052	2.383	0.017*	Accept
4	Perceived cost→ ISP compliance intention	-0.135	-0.136	0.043	3.146	0.002**	Accept
5	Perceived threat→ ISP compliance intention	0.050	0.055	0.046	1.099	0.272	Reject
6	Perceived effectiveness→ ISP compliance intention	0.285	0.285	0.077	3.704	0.000***	Accept
7	Self-efficacy→ ISP compliance intention	0.236	0.235	0.088	2.685	0.007**	Accept
8	Social influence→ ISP compliance intention	0.214	0.213	0.069	3.103	0.002**	Accept
9	ISP compliance intention→ ISP compliance behavior	0.872	0.873	0.018	47.466	0.000***	Accept

*p < 0.05;

**p < 0.01;

***p < 0.001.

Regarding statistical methods for evaluating the common method bias (CMB) issue, we adopted Harman’s single-factor test, full collinearity assessment, and the correlation matrix approach. Firstly, Harman’s single-factor test demonstrated that a single factor explained 32.243% of the variance, which was less than the threshold of 50% (Podsakoff et al., 2003). Secondly, the variance inflation factor (VIF) values (1.2 ≤ VIF ≤ 3.3) were lower than the threshold of 3.3 (Kock, 2015). Thirdly, correlations were assessed between the main constructs and were found to be less than 0.90 (Bagozzi et al., 1991). Therefore, these results suggest that CMB is not a serious concern for this study.

## 7 Discussion

The analysis results are shown in Figure 2 and summarized in Table 6. Of the nine hypothesized paths, three were found to be significant at a 0.001 level. The results confirmed the positive association between “technology characteristics” and “task–technology fit (TTF)” (β = 0.695; *t* = 17.819; *p* < 0.001). Therefore, H2 was supported. This can be interpreted as meaning that technology characteristics are found to be a key predictor for the TTF factor. The results provide evidence that information systems’ availability, accessibility, and technical consulting have an impact on target tasks regarding the ISP. In other words, the technology characteristics of information systems used by employees affect their task needs when reading and understanding the ISP. “Perceived effectiveness” had a positive relationship with “ISP compliance intention” (β = 0.285; *t* = 3.704; *p* < 0.001), which supported H6. This result could be interpreted as meaning that employees’ belief in their organization’s ISP effectiveness influences their ISP compliance intention. In other words, the more likely it is that the requirements of the ISP can effectively guard the organization against security threats, the more motivated organization members will be to comply with the ISP and encourage security behaviors. The results indicated that “ISP compliance intention” was positively related to “ISP compliance behavior” (β = 0.872; *t* = 47.466; *p* < 0.001), thus supporting H9. In other words, behavioral intention has a significant role in shaping compliance behavior. Similarly, Feng et al. (2019) and Siponen et al. (2014) provided evidence that the intention to comply with the ISP had a positive and significant impact on compliance behavior.

Furthermore, four hypotheses were found to be significant at a 0.01 level. “Task characteristics” were positively related to “TTF” (β = 0.136; *t* = 2.992; *p* < 0.01). Therefore, H1 was supported. This finding argues that task characteristics are essential predictors for specifying the suitability of the technology that needs to be applied to perform specific tasks. The result confirms that reading and understanding the ISP is an interdependence activity with others in the organization which, in turn, affects TTF. “Perceived cost” had a negative relationship with “ISP compliance intention” (β = -0.135; *t* = 3.146; *p* < 0.01), which supported H4. This result is interpreted to mean that the higher the ISP compliance cost, the less that employees will comply with the policy. This cost involves effort, time, money, etc. Decreasing the cost of compliance is essential to encourage compliance behavior by employees. One example of an ISP practice is changing passwords monthly, with employees considering it to be time consuming to create a non-similar password every time, which may lead to ignoring this practice.

The results also indicated that “self-efficacy” and “social influence” were positively related to “ISP compliance behavior” (β = 0.236; *t* = 2.685; *p* < 0.01; β = 0.214; *t* = 3.103; *p* < 0.01, respectively). These results supported H7 and H8. Self-efficacy is necessary for information security management as it contributes to the capability driver of behavior (Chen et al., 2016). The interpretation of this hypothesis could be described as meaning that employees’ beliefs about their self-efficacy towards ISP compliance affect their ISP compliance intention. With self-efficacy used by employees to evaluate their capacity to adhere to the ISP, organizations need to provide their employees with compatible training and the necessary technical skills to enrich their self-efficacy so they feel they are able to carry out ISP compliance (D’Arcy and Lowry, 2019; Ryutov et al., 2018; Hwang and Seo, 2025). Moreover, the members of social networks influence employees in their intention toward ISP compliance. This finding is aligned with the TTAT which posits that employees’ behaviors are definitely inspired by other people in terms of adopting safeguarding measures to avoid IT threats (Liang and Xue, 2009; Zhang and Pang, 2025). Ifinedo (2014) argued that employees who believe that ISP compliance benefits their colleagues and others in the organization will be more likely to perform a compliance behavior in their work.

Moreover, one hypothesis was found to be significant at a 0.05 level. “TTF” had a positive relationship with “ISP compliance intention” (β = 0.125; *t* = 2.383; *p* < 0.05), which supported H3. The findings show that when employees perceive that their information system fits their tasks regarding the ISP, this, in turn, impacts on employees’ ISP compliance intention in their work. In other words, employees who believe their organization’s information system fits their ISP tasks will be more likely to comply with the policy. Kim et al. (2020) assumed that the fit between the technology used by the organization and its ISP will influence the organization’s information security management. Hence, this fit influences ISPC behavior by employees.

However, a positively insignificant relationship existed between “perceived threat” and “ISP compliance intention” (β = 0.050; *t* = 1.099; *p* > 0.05), thus not providing support for H5. This finding is similar to Liu et al. (2020b) who confirmed that the path from perceived threat to compliance behavior is not significant. One possible explanation for the result is that perceived threat is measured from the organizational perspective; thus, it is not considered a main concern for employees (Liu et al., 2020b). What this finding suggests is that employees’ perceptions about information security threats are not truly significant to the ISPC guidelines.

The statistically non-significant relationship between perceived threat and compliance intention demonstrates the empirical limits of traditional, fear-based compliance approaches; heavy-handed scare tactics often decouple risk perception from actual behavior by creating systemic security stress and technostress rather than motivation. When employees are continuously inundated with alarmist corporate warnings, they experience severe security fatigue, causing them to cognitively tune out threat signaling to prioritize daily operational deadlines. Consequently, this predictive failure highlights a critical need to transition toward a human-centered cybersecurity governance paradigm—one that abandons weaponized anxiety and instead fosters policy adherence by maximizing technology usability, building individual self-efficacy, and establishing a supportive, risk-aware organizational culture.

## 8 Contributions

This section presents the study’s contributions to the research and practice.

### 8.1 Theoretical contributions

This study’s findings have several theoretical implications for information security policy research. Firstly, the current study responds to the call by Davison and Díaz Andrade (2018) to regard non-Western contexts in information systems research where the cultural and behavioral features vary from those of the Western world. Actually, this study provides a valuable direction for the ISPC research which, to date, has not been well studied in Saudi Arabia. Few researchers have investigated the ISP context in Saudi Arabia (Alzahrani et al., 2018; Thakur et al., 2016; Zakarya, 2018). Thus, this study provides a novel theoretical model which has never previously been empirically examined in such an environment.

Secondly, previous studies have considered the impact of human and organizational theories on shaping ISPC behavior, whereas they have failed to analyze the impact of technology-related behavior theories (Alassaf and Alkhalifah, 2021; Hengstler et al., 2023; Vedadi et al., 2024; Zhang and Pang, 2025). Therefore, this study fills the research gap by investigating the role of the TTAT and the TTF model in shaping employees’ ISPC behavior. Most significantly, this study develops and validates the TTF construct within ISPC research. This is considered to be the first such attempt in ISPC research and proposes that the fit between the technology used and the tasks regarding the ISP could be a significant influencing factor in ISPC behavior.

Thirdly, this study tests the TTAT in the light of security behaviors, as suggested by Liu et al. (2020b) and Zhang and Pang (2025). In that study, the TTAT was applied in an environment that mandates security behaviors, while the TTAT was examined as it applied to individual users in their homes (Liang and Xue, 2010). Therefore, the current study extends the well-established view of the theory by applying it in a work setting.

Fourthly, few prior studies have used the PLS Predict function (Koohang et al., 2021) to evaluate the model’s explanatory power; the current study, however, applies this function to increase the usefulness of the model in other environments. Finally, this study presents proof of the efficacy and suitability of the TTF model, the TTAT, and the TPB in measuring employees’ ISPC behavior. The research results provide a new insight into studying potential ISPC factors especially those related to technology-related behavior theories, with these having been considered outside the research context. Furthermore, this study also enhances the growing body of research that has studied information security behavior, as well as complementing previous efforts in the field.

### 8.2 Practical implication

The study’s results have several practical implications for organizations concerned about employees’ ISP compliance. Firstly, in light of the discovered impact of TTF on ISPC behavior, organizations’ management should provide detailed objectives of their policies in order to adopt information systems compatible with these policies. They should also include employees in decision making throughout the development of these systems. Information technology specialists should play a key role in ISPC by assisting employees with how information systems should be used in different tasks. In addition, with the rapid development of new technologies and systems, organizations’ information systems should be compatible and to fit their ISP’s requirements. Generally, organizations’ management should improve information systems to fit the various information security tasks, with this possibly improving their employees’ information security behavior.

Secondly, this study’s findings reject the view that perceived threats are affecting ISPC, which should be considered a serious concern for organizations’ management. Therefore, management should constantly re-assess employees’ perceptions of information security threats, explaining the potential extent of the damage caused by these threats (Siponen et al., 2014). Professional security education, training, and awareness (SETA) programs on such threats could be a beneficial option.

Thirdly, the significant findings in this study of the impact of ISP effectiveness on ISPC confirm that an effective SETA program is crucial to achieve ISPC enhancement. A SETA program is recommended to provide knowledge to employees about the effectiveness of an ISP. As Kim et al. (2014) suggested, a SETA program that teaches employees that the ISP is effective in addressing the organization’s information security is more important than enforcing information security techniques. Thus, the organization should provide an education and training program, and make it consistently available and easy to reach until employees ultimately adopt compliance behavior.

Fourthly, given the importance of self-efficacy to employees’ ISPC behavioral intention, managers should pay heed to helping employees improve their confidence about their capability to comply with security policies. Organizations’ management should render employees able to deal with emerging security technologies by themselves, and enhance the skills that they need to protect organizational information system assets (Ifinedo, 2014). Studies have argued that when employees have enough IT skills, this makes them much more confident in complying with the required regulations and policies (Liu et al., 2020b; Hengstler et al., 2023; Vedadi et al., 2024). Therefore, efforts should be channeled toward employees when organizations want enhanced compliance.

Finally, while the study’s findings provide evidence that employees are influenced by their social environment, creating a culture that supports security behaviors and activities should be a priority for organizations’ management. This can be achieved through an environment that enables employees to learn organizational values and the significance of the ISP through socialization with their co-workers. Identifying compliant behavior within the organization, such as sharing the best security performance either individually or within workgroups will enhance ISP compliance (D’Arcy and Lowry, 2019; Zhang and Pang, 2025). As a result, the current study’s findings also assist security managers and IT professionals to design their information security policies.

To optimize compliance, organizations must transition from fear-based enforcement to human-centered governance focused on policy usability, targeted training, and culture. Since Task-Technology Fit (TTF) drives compliance while Perceived Cost inhibits it, managers should treat security as a user-experience (UX) challenge by deploying frictionless configurations—like single sign-on (SSO) and automated conditional access—that integrate seamlessly into daily workflows. Furthermore, because abstract threat perceptions fail to motivate behavior, Security Education, Training, and Awareness (SETA) programs should abandon generic fear appeals and instead utilize action-oriented micro-learning modules that build employee self-efficacy. Finally, leveraging the power of social influence, management should cultivate an organic security culture through peer-led “Security Champions” programs, transforming policy adherence from a burdensome administrative task into a shared organizational value.

## 9 Research limitations and future work

This study has experienced some limitations that could be opportunities for future research. Firstly, the collected data were in the form of responses to a self-reported survey. This could result in possible sources of bias, such as socially desirable responses or even exaggeration or misunderstanding of measurement items by respondents that may impact on the data analysis and the generalizability of the results (Podsakoff and Organ, 1986). Future research may consider data collection methods other than the self-reported survey. A qualitative methods approach, such as interviews or focus groups, may yield better generalizability of results and provide further insights into ISPC research.

Furthermore, although the sample size of N = 288 enterprise participants satisfies the minimum statistical power thresholds required for variance-based Structural Equation Modeling (PLS-SEM)—comfortably exceeding both the traditional “10-times rule” and modern power analysis recommendations (Hair et al., 2021)—it remains somewhat restricted given the structural complexity, dual-stage appraisal mechanics, and multi-construct architecture of the integrated framework. While PLS-SEM is robust against smaller sample sizes in complex networks, future research should utilize larger, stratified data cohorts to further minimize potential parameter estimation bias, enhance statistical power, and cross-validate the structural path coefficients.

Secondly, while this study measures compliance behavior using a survey, other studies have used scenario-based surveys (Hong and Furnell, 2022; Lowry and Moody, 2015; Moody et al., 2018) to measure this behavior. These results therefore cannot be compared with each other. Therefore, scenario-based surveys should be used in the future to provide greater accuracy and to validate results. Thirdly, the largest group of respondents (24.3%) was in the education sector, followed by government employees with 23.6%. These proportions could have an impact on the results. Employees’ ISPC is more crucial in the finance, telecommunications, and information technology sectors, with these sectors more sensitive in dealing with data than other sectors. Therefore, future research should focus more on these sectors. Fourthly, the task characteristics scale was composed of only two items which might hinder the reliability of the construct as a scale in future research. Despite two-scale items being accepted for reliability, future research should try to include more items in this scale to enhance its reliability. Finally, while this study analyzed some dimensions of fit, future research may provide additional dimensions of fit.

## 10 Conclusion

The main objective of this study was to determine and examine the factors influencing employees’ ISPC behavior. The current study claims that this area of research could benefit from technology-related behavior theories. Through integrating the task–technology fit (TTF) model, the technology threat avoidance theory (TTAT), and the theory of planned behavior (TPB), the current study developed a research model to better understand the factors that could impact on employees’ ISPC behavior. This study aimed to investigate the relationship between these theories and ISPC behavior. The model was developed and then empirically tested using SmartPLS software. The data were collected from 288 employees working in either public or private organizations in Saudi Arabia. The results showed that the ISP, task characteristics and technology characteristics significantly influence TTF, which again influences an employees’ ISPC intention. The current study’s findings highlight the fact that ISPC intention is negatively influenced by the perceived cost of compliance. The results also revealed that perceived effectiveness of the ISP, self-efficacy, and social influence affect employees’ ISPC intention. However, perceived security threat was found to be insignificant for employees’ ISPC intention. Moreover, employees’ ISPC intention had a significant impact on their compliance behavior with these policies. The theoretical and practical implications of this study were discussed, along with the study limitations and directions for future research.

## Data Availability

The raw data supporting the conclusions of this article will be made available by the authors, without undue reservation.
