# Supplementary material for: Determinants of information security policy compliance: integrating task-technology fit and threat avoidance appraisals in the digital workplace
Source: Front Psychol. 2026 Jul 22;17:1861737. doi: 10.3389/fpsyg.2026.1861737 (PMC13437465; doi:10.3389/fpsyg.2026.1861737)
Supplement: Supplementary file 1 [file Table_1.docx]

## **Appendix : Items Development**

| **Construct** | **ITEMS** | | **Source** |
| --- | --- | --- | --- |
| Task Characteristics  (TC) | Task interdependence | TC1: When I read the information security policy, I often have to participate with others to accomplish the policy goal  TC2: Reading and understanding the information security policy has a great impact on others in my organization. | (Baas, 2010; Goodhue & Thompson, 1995; Morgeson & Humphrey, 2006) |
|  | Task non-routineness (Variety & difficulty) | TC3: While reading the information security policy, I regularly encounter difficult stuff that I don't immediately understand.  TC4: While reading the information security policy, I often need to perform multiple tasks to understand  TC5: Overall, reading and understanding the information security policy takes a long time for me. |  |
| Technology Characteristics (TNC) | Integrated, Common Systems | TNC1: There are many different information systems in my organization, each system has its own data. So, it's hard to identify which one to use in a given situation. | (Goodhue, 1995; Goodhue & Thompson, 1995) |
|  | Workstation Penetration | TNC2: PCs or Tablets are available for accessing the data that I need on the information systems. |  |
|  | Assistance Ratio\ Decentralization of Assistance | TNC3: I am satisfied with the level of technical consulting expertise I receive from the information technology department.  TNC4: Assisters are available when I need quick help with information systems problems. |  |
|  |  | TNC5: My organization offers me applications and programs that help to adapt the information security policy.  TNC6: My organization has a digital platform to explain the information security policies by some digital images, audio, and videos. | Self-developed |
| Task–Technology Fit (TTF) | Ease of Use\ Training | TTF1: It is easy to learn how to use the information systems that I need.  TTF2: I am getting the training I need to be able to use my organization's information systems. | (Goodhue & Thompson, 1995; Kim et al., 2020) |
|  | Systems Reliability | TTF3: The information systems that I used are subject to frequent system problems and crashes. |  |
|  | Production Timeliness | TTF4: my organization’s information security policy is performing well without negatively affecting the organization’s work interruption and productivity. |  |
|  | Compatibility | TTF5: The information system that I used is appropriate for my organization's information security policy requirements. |  |
| Perceived Cost (COST) | COST1: Complying with the requirements of the information security policy is takes a considerable amount of time and effort for me.  COST2: Complying with the requirements of the information security policy is burdensome for me.  COST3: Complying with the requirements of the information security policy is costly for me.  COST4: There is too much overhead associated with the adoption of information security policy.  COST5: Enforcing information security policy may cause distrustful relationships among employees. | | (Bulgurcu et al., 2010; Han et al., 2017; Liu et al., 2020) |
| Perceived Threat (PT) | PT1: IT security issues pose a threat to my organization.  PT2: The trouble caused by IT security issues threatens my organization.  PT3: IT security issues are dangerous for my organization.  PT4: It is terrible if my organization is confronted with IT security issues  PT5: It is risky to use my organization’s information system to work in case of IT security issues. | | (Liang & Xue, 2010; Liu et al., 2020) |
| Perceived Effectiveness (EFF) | EFF1：Complying with the information security policy would be useful for my organization to detect and eliminate IT security issues.  EFF2：Complying with the information security policy would enable my organization to protect information systems from IT security issues.  EFF3：Complying with the information security policy would enable my organization to detect and eliminate IT security issues faster.  EFF4：Complying with the information security policy would enhance my organization's effectiveness in searching and eliminating IT security issues.  EFF5: Complying with the information security policy would make it easier for my organization to detect and eliminate IT security issues. | | (Liang & Xue, 2010; Liu et al., 2020) |
| Self-Efficacy (SE) | SE1: I have the necessary skills to fulfill the requirements of the information security policy.  SE2: I have the necessary knowledge to fulfill the requirements of the information security policy.  SE3: I have the necessary competencies to fulfill the requirements of the information security policy.  SE4: I would be able to follow most of the information security policy even if there was no one around to help me.  SE5: I can follow the information security policy if someone tells me what to do as I go along. | | (Bulgurcu et al., 2010; Liu et al., 2020; Sikolia et al., 2016; Siponen et al., 2014) |
| Social Influence (SI) | SI1: People who influence my behavior think that I should comply with the information security policy.  SI2: People who are important to me think that I should comply with the information security policy.  SI3: The senior management of my organization has been helpful in complying with the information security policy.  SI4: In general, my organization supports information security policy compliance. | | (Venkatesh et al., 2003) |
| ISP Compliance Intention (CI) | CI1: I intend to follow the information security policy in my organization.  CI2: I will possibly comply with the information security policy to protect the organization’s information systems.  CI3: I am sure I will follow the information security policy in my organization.  CI4: It is clear that I will follow the information security policy in my organization. | | (Ifinedo, 2012; Kim & Han, 2019) |
| ISP Compliance Behavior (CB) | CB1: I always comply with the requirements of the information security policy of my organization during my work.  CB2: I never violate the information security policy of my organization.  CB3: I recommend others to comply with information security policy.  CB4: I assist others in complying with information security policy.  CB5: I usually ignore the information security policy of my organization for convenience. | | ( Liu et al., 2020; Siponen et al., 2014) |
